# A Case of Extra-Uterine Fœtation

**Published:** 1828-05

**Authors:** 


THE LONDON
Medical and Physical Journal.

N° 351, VOL. LIX.] MAY, 1828. [N° 23, *New Series.*

For many fortunate discoveries in medicine, and for the detection of numerous errors, the world is indebted to the rapid circulation of Monthly Journals; and there never existed any work, to which the Faculty, in Europe and America, were under deeper obligations, than to the Medical and Physical Journal of London, now forming a long, but an invaluable, series.—RUSH.

### ORIGINAL PAPERS,

AND

### CASES OBTAINED FROM PUBLIC INSTITUTIONS AND OTHER AUTHENTIC SOURCES.

### EXTRA-UTERINE FŒTATION.

*A Case of Extra-Uterine Fœtation.* By — TILT, Esq.

SOPHIA LICKISH, æt. thirty-seven, was twice married; bore four children to her first husband, and, six years after her second marriage, again became pregnant. During her former pregnancies nothing unusual occurred, and her confinements were reported to have been short and easy; and nearly three months of the fifth had elapsed before any accident gave rise to untoward symptoms. While in the country, at the commencement of September 1827, she was exposed to unusual exertion in drawing water from a deep well, during which she felt as though something internal had given way; which was immediately followed by discharge of blood per vaginam, vomiting of bloody fluid, impaired appetite, and tenderness of abdomen. To these were shortly added retention of urine and constipation of bowels. Being no longer able to discharge the duties of her situation, she returned to her husband in town. Medical aid was now procured: her water drawn off with the catheter, and her bowels opened by medicine; which course of treatment was found necessary for a considerable time, when, the symptoms somewhat lessening in violence, fewer purgatives were required, and pressure above the pubes was found sufficient to empty the bladder.

Upon the 28th of January, 1828, I first saw her. She was in bed, to which she had been constantly confined since the accident. She was much emaciated, her general health very bad, and her strength nearly exhausted by frequent vomiting, a copious fetid discharge from the vagina, and incessant abdominal pain, which she compared to lingering labour-pains. She now considered herself more than seven months gone, and was unusually large. Although the symptoms which existed, when conjoined to the history of her case and her present appearance, left little doubt in my mind as to its real nature, yet, anxious to neglect nothing which might assist my diagnosis, I requested an examination, which was readily granted; but nothing could be satisfactorily ascertained. The os tincæ could be nowhere felt; no part of the child was recognisable; and a tumor of considerable size lay pressing against the sacrum. Two medical friends (Dr. H. Davies and Mr. Clark, 57, Lamb's Conduit-street,) were also allowed to examine her, but nothing more was elicited. These circumstances, viewed in connexion with the symptoms above mentioned, led us to conclude that the child was extra-uterine, and likewise to suspect retroversion of the uterus.

By the administration of small opiates, the irritability of her stomach was diminished, the intestines were kept open with castor oil, and her health gradually improved under a soothing plan of treatment.

Up to the 10th of February, nothing occurred of any importance; but, on the morning of that day, without any apparent cause, the vomiting returned; pressure above the pubes relieved the bladder with difficulty; her countenance became much altered; and the motions of the child, of which she had been sensible for the last three months, together with the vaginal discharge, now ceased.

At the suggestion of Dr. H. Davies, the opiate was conjoined with chalk mixture; and a blister was applied to the epigastrium, on account of the irritability of the stomach, which had again returned. Although the blister did not rise well, and the draught was rejected, she felt relieved. On the day following, effervescing draughts, occasionally administered, with opium and capsicum, kept the stomach quiet, and procured some rest at night. Her bowels acted freely this evening without medicine, and the next morning her urine flowed, for the first time, without pressure over the pubes. Notwithstanding, however, these slight signs of improvement, she continued gradually to sink until the 14th, when she died.

An inspection of the body being a great desideratum, the friends were consulted, and permission was accordingly obtained. Dr. H. Davies and Dr. Dill were present at the examination, in which I was assisted by Mr. Bell, a friend of the former gentleman.

*Post-mortem appearances.*—The skin was universally jaundiced; the feet slightly œdematous; the abdomen very much, but irregularly, enlarged, being considerably fuller on the left side; the mammæ were flaccid and contained milk; and the external parts of generation were as tumid as in natural cases of labour. By moving the hand carefully over the surface of the abdomen, the head of the child could be distinctly felt, lying a little above and to the left of the umbilicus, and one of its extremities (whether hand or foot could not be satisfactorily determined,) was easily recognised resting above and to the right of the head. By tapping the surface over these two points with the finger, fluctuation was very palpable; and the tenuity of the intervening integuments, apart from the position of the fœtus and other corroborating symptoms, rendered the nature of the case completely evident. The arch of the colon was distinctly traced running across the epigastrium, immediately above the fœtal mass, and the abdominal covering was, by its pressure outward, moulded into a form somewhat similar to itself.

Upon making a longitudinal incision through the parietes of the abdomen, it was found that a large quantity of fluid lay in the peritoneal sac, and that the dissection could not be prosecuted with the necessary precision until it was removed. An opening was, therefore, made in the lowest part of the right lumbar region, through which much bloody fluid escaped, and the remainder was extracted from above by a sponge, the whole amounting to four or five pints.

A transverse incision was now made, and the flaps everted with considerable difficulty, as the peritoneum was glued somewhat closely to that portion of the membranes lying over the child's head.* The contents of the abdominal and pelvic viscera being now fairly exposed, a tolerably minute inspection furnished us with the following detail:

The peritoneum was highly inflamed, very dark, and considerably thickened. The small intestines were pushed up into the bosom of the diaphragm, and the omentum, which was wasted to a mere shred, lay reflected over the stomach. The liver was usual in size, but its peritoneal envelope, in common with that of the other viscera, was as much changed in texture as in aspect: nothing morbid, however, could be discovered in its internal structure, and the gall-bladder and biliary ducts appeared natural.

The uterus was seen lying obliquely across the pelvis, with its fundus in the right lumbar region, and its os tincæ rather above, and to the left of the symphisis pubis. Its size was above that of its ungravid state: it felt tolerably firm, and was externally very dark coloured. Between it and the intestines, principally in the umbilical and left lumbar regions, lay the fœtus involved in its membranes; the placenta being exterior to, and betwixt them and

* I think it is more than probable that, had the woman survived much longer, the abdominal integuments would have suppurated at this point, allowing the child to escape in detached pieces. See American Magazine for 1746, where a case is given in which several parts of a child came thus away, after remaining in the abdomen for several years, during which the woman bore six children: she, however, died. See likewise Journal de Med. Chir. &c. tom. xiv. page 443, for a case in which a complete skeleton was removed from the abdomen, through an aperture in the umbilical region formed by sloughing. The patient perfectly recovered, and ever after menstruated through this opening.

the arch of the colon. A fillet of fibro-ligamentous substance, about an inch and half in width, ran down from the inferior surface of the arch of the colon over the membranes, and was inserted into the superior edge of the left ligamentum latum. This band being divided and turned aside, the subjacent membranes were carefully opened, and the fœtus exposed to view. It was smaller than the average of full-timed children, but must at least have been eight months old. It lay upon its left side, with its lower extremities stretched over the sacral promontory into the posterior pelvic cavity; and its head, which was somewhat reclined, resting upon its left arm. Its face thus looked towards the diaphragm, and its chest was presented to the left side. The left side of its head and face was flattened, in consequence of pressure upon the arm, but, excepting this accidental deformity, it seemed perfect and well-shaped. As the motions of the fœtus had not been perceived for five days previous to the woman's dissolution, it was believed that the child had been so long dead; but, from abrasion of the cuticle and other signs of decomposition, it is likely that this event happened earlier than was suspected. There was no more than two or three ounces* of fluid in the membranes, which was of a very dark colour, apparently from admixture of blood. The chorion and amnion were perfectly natural, differing in nothing from those formed in the uterine cavity; and exterior to these lay the sac,† or ventral uterus, within which the fœtal mass, excepting the placenta, was contained. The walls of this sac were about three lines thick, of considerable strength, perfectly smooth internally, and tenaciously adhering to the neighbouring parts. It was attached to both the broad ligaments, descended along the posterior surface of the uterus in company with the subjacent peritoneum, was reflected over the sacral promontory, adhered in its ascent to the sigmoid flexure of the colon on the one side, and to the psoas muscles on the other, rose upon the lumbar vertebræ, swept over the internal surface of the placenta, and, turning a little above the umbilicus, ran down over the membranes to the ligamenta lata. It was nowhere connected with the chorion, and yet no space intervened between them. Above it, and considerably to the left side, lay the fillet before described.

The placenta was of enormous size,‡ and very extensively attached. Its width was greater than usual, it was five inches in thickness, and could not have weighed less than five pounds. In

* The average quantity of water contained in the amnion in ordinary cases is about two common pints.

† A case is recorded in the seventh volume of the New-York Medical Repository, page 221, where a sac of a very similar character was found, and the anterior portion of which, as in the present instance, adhered to the peritoneum lining the muscles of the abdomen.

‡ In cases of extra-uterine fœtation, the placenta is much thinner, and, although more extended than uterine placentæ, is considerably lighter and less bulky. I am not aware that there is any case recorded in which the placenta equalled the present, either in weight or size.

structure it differed little from those met with in ordinary cases; perhaps, it was somewhat firmer. It adhered with great tenacity to the sac, excepting in the inferior part on the right side, where it was detached for a few inches, the intervening space being filled with coagulated blood.* Its supply of this fluid must have been very liberal, as its connexion with blood-vessels was very extensive. The right, and especially the left mesenteric and emulgent vessels seemed to be the main fountains from which nutriment was drawn for the fœtal system; but, besides these, there were many minor sources, since it adhered throughout to the inferior surface of the arch of the colon, a great part of the mesentery and meso-colon, to portions of the small intestines, and two or three of the superior lumbar vertebræ. It lay across the spine, but unequally, the greater part of it being on the left side; and the surface, by which it was connected with the mother, faced the diaphragm, from which it was separated by the intestines which had been forced up into the bosom of this muscle.

Nothing could be discovered amiss in the structure of the uterus, which was about the size of one in the fourth month of pregnancy.† Externally it was inflamed, and presented the same darkened appearance with the abdominal viscera. We have already stated that the sac adhered to its posterior surface, and, upon separating this membrane, for which considerable force was necessary, slight vascularity appeared behind and a little to the left of the right lateral ligament; but there was not a vestige of cicatrix, nor did any thing exist to sanction the idea of rupture of this organ. The os tincæ was dilated, and two fingers could be admitted with ease. The cervix appeared slightly vascular, but the internal surface of the fundus was pale and smooth. The right fallopian tube was pervious, of natural size, and complete; but there was no right ovarium to be perceived. The left ovarium was small, and contained several vesicles. The left fallopian tube was somewhat enlarged, and about the middle of its superior surface, near to the insertion of the fillet before mentioned, its coats were ulcerated for an inch and a half.‡ Upon either side of this opening it was pervious, and its calibre throughout larger than that of the right. The vagina was quite healthy.

Several questions of importance must suggest themselves to the mind on reading the above detail; and the first in interest undoubtedly is, how did the fœtus make its way into the abdominal cavity? Did the ovum remain in the ovarium,

* The hemorrhage which resulted from this partial detachment of the placenta had, no doubt, accelerated the death of this woman; and it was the blood, then effused, that darkened the fluid which before lay in the peritoneum.

† It is generally found more or less enlarged in such cases.

‡ A case is detailed by Mr. Tucker, in which the left fallopian tube was ruptured about the same part; and the death of the woman proceeded from partial detachment of the placenta.

and did the ovarium, by gradually enlarging, constitute the sac in which the fœtal system was contained? Or did the ovum, in leaving the ovarium, fall into the abdomen; not being grasped by the fallopian tube? Or did the ovum, during its passage through the fallopian tube, escape into this cavity? Or, finally, did it reach the uterus, and, by retroversion of this organ, constitute one of those cases described by Dr. Merriman?*

It will be admitted, I presume, that the answer must be found in some one of these suppositions; and, although there are many circumstances of obscurity accompanying the history as well as the dissection of the case, and there may be arguments advanced in support of each, several reasons have induced me to conclude that it was through the left fallopian tube the ovum escaped into the abdomen.

We have no reason to believe that its escape was occasioned by the corpus fimbriatum neglecting to embrace it on its departure from the ovarium; and there exists no evidence to show that there ever was retroversion in this case. During the first six months of pregnancy, no examination per vaginam was made; and, although retention of urine and constipation of bowels are presumptive of this misplacement, yet these symptoms did not occur until the third month of pregnancy, when the fœtus, membranes, and placenta had evidently arrived at an unusual size, and, by their position behind the enlarging uterus, will satisfactorily account for their appearance. It is true that several medical gentlemen had examined her some weeks before her dissolution, and suspected retroversion; but the absence of the os tincæ, and the tumor that pressed upon the rectum, the only circumstances on which our diagnosis was founded, were otherwise explained by inspection after death, when the uterus was found in a position the reverse of that assumed in retroversion.†

The absence of the right ovarium was the only fact favorable to the supposition that this was a case of ovarium pregnancy; and, independent of the impossibility of determining whether or not it did ever exist, I think there are several circumstances tending to show that this solitary fact cannot be regarded as conclusive.‡ The fœtus lay almost entirely on

* See his Dissertation on Retroversion.

† It is extremely difficult, perhaps impossible, in some such cases, to determine when retroversion exists, and not a few have experienced the same difficulty with ourselves. See Medical Commentaries, vol. xx. page 254; London Medical Journal, vol. v. page 96; Medical and Physical Journal, vol. xi. page 293; Transactions of a Society, &c. vol. ii. page 287.

‡ Ovarian cases are not very common, and it seldom happens that the ovary reaches a large size. "It either bursts early, or inflammation and the left side, and it was the right ovarium that was absent; —the adhesions of the sac, in which it was contained, were much more numerous on the left than on the right side;—the fillet which bound down the membranes was inserted into the left fallopian tube, near to the point of its ulceration, and lay entirely in the left side; and the greatest portion of the placenta was attached to viscera belonging to the left side. Besides, had this been an ovarian case, why did the woman pass the first three months of pregnancy in health and ease; and why did symptoms of disorder, when they did appear, come on instantly and with violence? Had the ovum never left the ovarium, the gradual increase of this latter organ would have prevented the sudden approach of constitutional derangement; and, although the disturbance occasioned might have been ultimately great, it would not have commenced with violence.

We are, therefore, inclined to believe that the ovum escaped, during its passage through the left fallopian tube, into the abdominal cavity, and was gradually invested with membranes and a false uterus. Could it be shown that the right ovarium had never existed in this woman, this view of her case would be demonstrably true, seeing that the ovum must have been impregnated in the left ovarium, and that the left fallopian tube was found ruptured. But, as we have nothing beyond presumptive evidence that such was the case, although highly probable, it cannot be considered certain.

The position of the child, and the strong and numerous adhesions of the sac in the left side,—the easy and natural course of her pregnancy for the first three months,—the sudden supervention of untoward symptoms,—the feeling of something having given way internally while using violent exertion, instantly followed by derangement of the whole system, and a bloody discharge per vaginam,—the continuance of this derangement, with increasing violence, from this period to that of her decease,—the fact that the ovum does not leave the ovarium until six weeks or two months after impregnation, and the near correspondence of this period with that which elapsed prior to the accident,—the ruptured condition of the left fallopian tube, and the formation of the fillet at the margin of the rupture,—the appearance of the sac being that of a false membrane, and not of a bag covered with the peritoneum,—the presence of the greatest part of the placenta in the left side, and the absence of the right ovarium; abscess take place, or the fœtus dies and is converted into a confused mass, or it excites dropsy of the ovarium." (Burn's *Midwifery*, page 193.)

are the principal circumstances by which we have been influenced in forming our opinion. Whether the ovum lodged for some time in the tube before the accident, or had merely proceeded so far through it when the accident occurred, are circumstances difficult to determine. Perhaps, the larger cavity of the left tube, and the slight difference* which existed between the period that elapsed from the commencement of pregnancy until the injury was sustained, and that generally occupied before the ovum leaves the ovarium for the uterus, are circumstances favorable to the first supposition, yet cannot be deemed sufficient to establish the point.

That there would have been some discharge of blood after the accident might have been expected, but that this discharge should have been continued unto the period of the child's death, when it ceased, appears strange and inexplicable. Was this the menstrual fluid,† or some morbid accidental discharge? If the former, the ordinary course of nature in such cases was reversed; for, during the life and growth of the child the menses are discontinued, but return after its decease; and, if it were a morbid product, where was its source? Was it secreted by the vagina, or did it come from the uterus? or could it possibly have drained from the abdomen through the ruptured tube, as a part of that fluid, some of which was taken out of it after death? The internal surface of the uterus and vagina, excepting a small portion of the cervix of the former, was pale, and betrayed no symptoms of having given rise to any such secretion, and the external characters of both these fluids were very similar; but, admitting that they were the same, we are furnished with no reason why the discharge was discontinued upon the death of the child, and, moreover, it is very doubtful whether fluid existed in the abdomen during all the time which elapsed from the occurrence of the accident.‡

Indeed, the entire case is so perplexing and obscure, that almost every part of it may be considered a subject for dis-

* Although the period above specified is the average time spent by the ovum in the ovarium, yet it may certainly remain longer; but, supposing that two months of pregnancy had passed before its departure, a few days only would be the difference between that period and that which elapsed between conception and the date of the accident, which occurred some time during the third month.

† Cases are upon record in which the menses flowed for several months during pregnancy. (Vide BURNS, page 187.)

‡ From the inflamed condition of the peritoneum in this case, it is probable that the greater part of the fluid found in the abdomen after death was the effect of peritonitis; and it is not to be conceived that such a disease could have existed long prior to the decease of the woman.

cussion and difference of sentiment; and, acting under this conviction, while for reasons adduced we have defended the views now given, all the minutiæ of the case are laid before the reader, that he may have materials from which to draw his own conclusions.

